# Functional Connectivity and Volumetrics Improve Outcome Prediction for Deep Brain Stimulation in Parkinson’s Disease

**DOI:** 10.1002/mdc3.70108

**Published:** 2025-05-05

**Authors:** John R. Younce, Scott A. Norris, Mwiza Ushe, Joel S. Perlmutter

**Affiliations:** 1 Department of Neurology, University of North Carolina at Chapel Hill, Chapel Hill, NC, USA; 2 Department of Neurology, Washington University in St Louis, St Louis, MO, USA; 3 Department of Radiology, Washington University in St Louis, St Louis, MO, USA; 4 Departments of Neuroscience, Physical Therapy, Occupational Therapy, Washington University in St Louis, St Louis, MO, USA

**Keywords:** clinical prediction, deep brain stimulation, functional connectivity, Parkinson’s disease, volumetric MRI

## Abstract

**Background:**

Deep brain stimulation (DBS) targeting the subthalamic nucleus (STN) can effectively treat motor symptoms of Parkinson’s disease (PD). However, optimal patient selection remains challenging due to the inadequacy of outcome predictors. Most clinicians rely on levodopa response to predict DBS motor outcomes, yet previous studies have identified other MRI-based predictors including resting-state functional connectivity (FC) and volumetric measures.

**Objectives:**

To compare the predictive value of functional and volumetric MRI data with levodopa response alone for motor outcomes in STN DBS for PD.

**Methods:**

We analyzed 65 participants who underwent STN DBS for PD at Washington University in St. Louis. We used relaxed LASSO regression to select factors including clinical, volumetric, and FC measures to generate predictive models for relative changes in motor scores post-DBS and assessed cross-validated performance. We then compared the relative influence of the predictive factors with standardized regression coefficients.

**Results:**

Addition of MRI predictors (FC and volumetric) improved model fit and cross-validated model performance over levodopa response alone (levodopa alone: *R*^*2*^ = 0.191, RMSE 13.6; MRI + levodopa: *R*^*2*^ = 0.31, RMSE 12.6). Within the optimized model, aggregated FC and levodopa response exhibited the highest influence on motor outcome prediction.

**Conclusions:**

Including MRI-based predictors significantly enhances the prediction of motor outcomes in STN DBS compared to levodopa response alone, with FC predictors demonstrating the greatest influence in the optimized model. External validation studies are necessary to confirm the clinical utility of these predictors in routine practice.

Deep brain stimulation (DBS) of the subthalamic nucleus (STN) is an established and effective adjunctive therapy for motor symptoms of Parkinson’s disease (PD), particularly for reducing motor fluctuations and medication-refractory tremor.^1^ Despite its general effectiveness, patient outcomes vary widely across studies and even within most studies.^2–4^ This variability in combination with the invasive nature and significant cost of this procedure necessitate careful patient selection to maximize outcomes in comparison with inherent risks.^5,6^

The traditional metric used to evaluate potential response to STN DBS is response to a levodopa challenge, with early reports suggesting a high correlation between responses to STN DBS and levodopa.^7,8^ However, subsequent reports have favored a weaker and less consistent relationship between DBS and levodopa responses, with one study identifying serious mathematical errors underlying much of this supposedly robust relationship.^9–11^ Numerous studies have attempted to characterize additional predictors of DBS response, with varied success. Age has been reported as a negative prognostic factor for STN DBS, although other studies have more recently shown no difference in DBS response or complication rate based on age alone.^7,12,13^ Similarly, although tremor has been noted to respond particularly consistently to STN DBS, tremor-dominant and postural instability-gait difficulty PD subtypes do not appear to differ significantly in their overall motor response.^14,15^

Limitations in the ability of clinical characteristics to predict DBS response has led to efforts to use neuroimaging tools to relate DBS responsiveness with variations in brain structure and function. Various volumetric MRI studies have reported associations between structural brain differences and DBS outcomes using both cortical and subcortical volumetric measures, including thalamic and ventricular volumes,^16^ midbrain volumes,^17^ visuo-motor cortical thickness,^18^ and superior frontal and paracentral cortical thickness.^19^ Furthermore, alterations in brain function may be examined using resting-state functional connectivity MRI (rs-fcMRI), which allows the assessment of synchronized functional systems within the brain spanning distant neuroanatomical areas.^20^ PD patients have clear alterations in functional connectivity (FC),^21^ which also vary between PD subtypes^22,23^ stages of progression,^24,25^ and which may or may not be affected by dopaminergic treatment.^26,27^ DBS alters neuronal activity at both a local and a network level, possibly restoring network alterations caused by PD.^28,29^ Examination of FC in DBS patients also has taken several forms including hypothesis-driven analysis of FC between seeds identified by blood flow responses to DBS,^30^ analysis of regional neural fluctuations differing between PD and control subjects,^31^ and network-level analysis at a hemispheric level.^32^ However, to date, no comprehensive predictive model for DBS response integrating clinical predictors with both functional and structural MRI-derived predictors has been developed.

A comprehensive model that can predict motor outcomes following STN DBS for PD using a combination of pre-operatively available clinical and MRI-based predictors has the potential to enhance clinical efficacy by improving patient selection. Restricting predictors to data available pre-operatively (ie, during the DBS evaluation process) is crucial to the future utility of these models to perform clinical prediction of outcomes in clinical scenarios, thus excluding variables like electrode location and stimulation field as predictors. We thus aimed to evaluate the value of pre-surgical MRI-based measures, including those derived from FC and volumetric analysis, in comparison with levodopa response for the prediction of relative change in motor scores to STN DBS in PD. We also aimed to construct an optimized model from a comprehensive predictor set and test its overall performance.

## Methods

### Participants

This research was approved by the Institutional Review Boards at Washington University in St. Louis (initial data acquisition and analysis) and the University of North Carolina at Chapel Hill (subsequent data analysis). Participants were selected from a series of PD patients who had consented to participate in this study and planned to undergo bilateral STN DBS surgery at the Movement Disorders Center (MDC) at Washington University School of Medicine (WUSM). To be included, participants must have met UK Brain Bank criteria^33^ for diagnosis of idiopathic PD, placement of bilateral STN DBS between 2007 and 2017 (reflecting time period when this series of pre-DBS research MRI scans were collected), and adequate clinical and MRI data for analysis. Motor scores were assessed using the United Parkinson’s Disease Rating Scale part III (UPDRS-III). Patients were excluded from DBS surgery if they had metal or MRI-incompatible devices in the body, history of dementia or inadequate levodopa response of less than 30% change in UPDRS-III (except for medication-refractory tremor), structural brain abnormalities precluding safe electrode implantation, history of encephalitis, stroke, serious head injury or an inability to hold the head still. We excluded participants from analysis for one or more of the following reasons: inaccurate electrode placement (three participants, see “Neurosurgical procedure” below), did not undergo STN DBS for any reason (eight participants), patients with missing motor outcome data (six participants), and inadequate MRI data following rigorous parameters for motion censoring and quality control (39 participants, see “MRI methods” below).

### Clinical Evaluation

Patients who were considered clinical candidates for STN DBS for PD following evaluation at WUSM MDC by a movement disorders neurologist were screened for surgical contraindications and completed formal neuropsychological testing to exclude dementia. Prior to electrode implantation, we obtained UPDRS-III scores in the practically defined OFF-medication condition (at least 8 h following last dose of dopaminergic medication), then administered levodopa and obtained UPDRS-III scores in the ON-medication condition. These pre-surgical ON and OFF scores were used to calculate levodopa response. Additional preoperative clinical data collected included demographic information (sex, handedness), age at PD symptom onset and diagnosis, presence of key clinical features (dyskinesia, dystonia, freezing of gait, hallucinations, depression, anxiety, and cognitive impairment) on preoperative clinical evaluation, Mattis Dementia Rating Scale (DRS) and age-based percentile score, z-score for one of two depression scales (Geriatric Depression Scale or Beck Depression Inventory-II),^34,35^ preoperative Mini-Mental Status Exam (MMSE), and levodopa equivalent daily dose at time of electrode implantation (LEDD).^36^

Following electrode implantation, participants received standard of care postoperative DBS programming in addition to medical management for PD. Briefly, this included initial programming at approximately 30 days following electrode implantation, monopolar review of all electrodes for therapeutic window to determine optimal stimulation contact, monthly follow-up with stimulation adjustment until practical optimization was achieved, and gradual decrease of dopaminergic medications as needed to prevent side effects following DBS. All OFF-medication, ON-stimulation scores were collected for 12 months after DBS implantation. Average change in UPDRS- III was calculated over the postoperative period to mitigate the impact of random fluctuations and comprehensively represent overall postoperative condition. UPDRS-III inter-rater reliability was validated for all UPDRS-III raters on an annual basis via video-based testing.^37^

### Neurosurgical Procedure

All participants received bilateral electrode implantation in the STN using Medtronic 3389 leads. Electrode implantation was performed as previously described, including use of microelectrode recording and intraoperative sensorimotor testing of implanted electrodes to confirm accurate electrode placement, followed by postoperative CT.^38^ Electrodes were then localized and compared with a probabilistic atlas by merging patient anatomy with atlas space using a previously validated method.^39^ Electrode placements were considered accurate if within 2 mm of probabilistic STN or within cluster of electrode placements in posterolateral STN and border zone.^40^ Participants were excluded if bilateral accurate electrode placement could not be confirmed using this method.

### MRI Methods

#### Acquisition

We acquired MRI data prior to electrode implantation (median for included participants 12 days before DBS, range 1 to 295 days) using a 3 T Siemens MAGNETOM Trio system (Erlangen, Germany) and a 12-channel head coil. All sequences were obtained in the ON-medication condition to minimize the effect of movement (ie, tremor, OFF-state dystonia) and promote patient tolerance. Participants were asked to lie awake with eyes closed in the scanner and remain still. Patients were observed during scanning, and BOLD runs with visible body motion (including tremor and dyskinesia) or where the patient reported sleep were discarded. Sequences performed included a T_1_-weighted MPRAGE (TR = 2400 ms, TI = 1000 ms, TE = 3.13 ms, FA = 8°, 0.9 mm^3^ voxels), a T_2_-weighted fast spin echo (TR = 3200 ms, TE = 469 ms, FA = 120°, 0.9 mm^3^ voxels), and 1–4 rs-fcMRI BOLD EPI runs (TR = 2200, TE = 27 ms, 200 volumes per run, 7.3 min acquisition time).

#### Volumetric Processing

For volumetric analysis, structural MRI images were processed using FreeSurfer version 7.3 (https://surfer.nmr.mgh.harvard.edu/).^41^ Processing included cortical reconstruction and volumetric segmentation using both T_1_ and T_2_ structural MRI data. We performed visual inspection of segmentation to ensure quality control and made manual edits to correct small errors, while we excluded participants with globally poor quality segmentation. For participants with good quality segmentations, we extracted cortical volumes and thicknesses of total cortex and sensorimotor cortex (precentral, postcentral, and paracentral), as well as subcortical volumes of caudate, putamen, pallidum, thalamus, lateral ventricles and 3rd ventricle.

#### FC Preprocessing

We performed rs-fcMRI preprocessing and motion censoring per rigorous methodology similar to previously described methods using the 4dfp suite of tools (http://4dfp.readthedocs.io) and updated to the most current preprocessing pipeline.^42,43^ Briefly, sessions were corrected for odd-even slice intensity differences, normalized to a mode of 1000, and corrected for head movement within-run. Mean-intensity BOLD images were transformed to Talairach atlas space, and distortion correction was performed using synthetic field maps.^44^ We computed temporal masks for frame censoring using both DVARS and frame displacement (0.3 mm) criteria, then performed data demeaning and detrending and linear interpolation across censored frames. A component-based approach to nuisance regression was used, with components derived from white matter and CSF masks, as well as global signal and its first temporal derivative and six motion parameters. Bandpass filtering (0.005 to 0.1 Hz) was performed after nuisance regression to avoid reintroduction of filtered nuisance frequencies.^45^ Finally, data was smoothed using a 7 mm Gaussian blur, and temporal filters were applied to censor frames for analysis. Participants were excluded if at least 150 frames (5.5 minutes) were not retained following censoring using temporal masks.

#### FC Computation

A combined set of regions of interest (ROIs) for rs-fcMRI analysis were defined from two sources: (1) areas of significant blood flow response to STN DBS derived from a prior study^30^ (“DBS response ROIs”), and (2) a set of 300 ROIs divided into major cortical and subcortical resting state functional networks.^46^ ROIs were excluded if they experienced signal dropout in any participant, which resulted in the exclusion of three cerebellar ROIs, all of which were located at the inferior border of cerebellum and not included in FOV in three participants. We extracted pre-processed fMRI time series from each ROI and performed Pearson’s correlation between each ROI. To reduce dimensionality, we adopted four approaches: (1) FC between STN and each DBS response ROI, (2) FC between STN and composites of each resting state network, (3) Mean intra-network FC for each resting state network, and (4) principal component analysis (PCA) of FC between all ROIs, with the 10 principal components (PCs) with highest eigenvalues (representing 80% of total variance) used for analysis.

### Statistical Analysis

To compare the predictive value on DBS response of various data types, we divided predictors into four groups: (1) percent change in motor response to levodopa from pre-surgical ON/OFF medication testing (levodopa response), (2) additional clinical data, neuropsychological test results (see “Clinical evaluation” section), (3) volumetric MRI data, and (4) rs-fcMRI data. Volumetric predictors included each cortical and subcortical measure obtained based on segmentation (see “MRI Methods,” *Volumetric* processing). rs-fcMRI predictors included all predictors from each dimensionality reduction approach, including FC from STN to each DBS response seed and canonical resting state network, intranetwork FC composites, and 10 PCs (see “MRI methods,” *FC* computation). Based on prior work demonstrating that absolute preoperative levodopa response produces artificially high correlations with DBS response^11^ (analysis replicated for our data in Figs. S1 and S2), we restricted levodopa response data and other clinical data to percent change with respect to the UPDRS-III and its subcomponents when used as predictors of DBS response. As the most widely-used clinical predictor, levodopa response was considered the basis for comparison, and each additional predictor set was added to it. We also combined all data groups to create a potential predictor set for a comprehensive predictive model. Spearman’s correlations were computed between all potential predictors in the comprehensive model to evaluate for potential issues with multicollinearity (Supplementary File 1).

**Figure 1.**
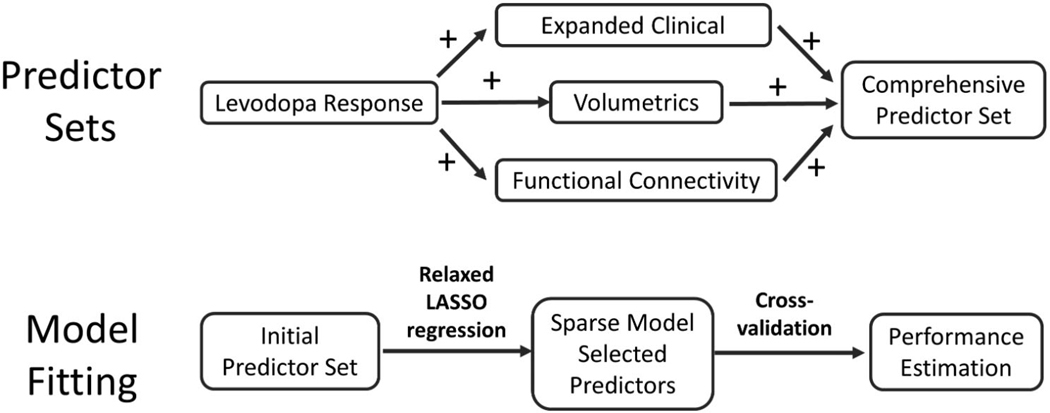
Model generation strategy. Predictor sets began with percent levodopa response, followed by adding one of three additional data types (expanded clinical, volumetries, functional connectivity). All data types were combined to create the comprehensive predictor set. Model fitting was performed for each predictor set independently, using crossvalidated relaxed LASSO to select a sparse set of predictors. Leave-one-out crossvalidation was used to estimate out-of-sample performance on each model for comparison between predictor sets.

We employed a relaxed least absolute shrinkage and selection operator (relaxed LASSO)^47^ approach using the *penalized* package^48^ in MATLAB 2022a (Mathworks, MA, USA) to select predictors and build models. In this method, regularized linear regression is performed using an L1 (LASSO) penalty to reduce coefficients of unimportant variables to zero, using minimized cross-validated deviance to estimate the penalty (*λ*). An additional parameter (*γ*) which “relaxes” this penalty on non-zero coefficients then is similarly tuned to generate an overall optimized model. This yields a sparse model including only a limited set of predictors from the pool of available predictors. Influence of interaction terms were also considered using package “glinternet” in R 4.2.3.^49^ For each predictor set, we first constructed a sparse model using relaxed LASSO. Use of LASSO mitigates the impact of multicollinearity on model performance and provides robust factor selection, although the selection of particular collinear variables should not be overinterpreted. *F* tests were performed using residual sum of squares (RSS) to evaluate significant differences between models, and Benjamini-Hochberg False Discovery Rate (FDR) was used to correct for multiple comparisons. We then performed leave-one-out cross-validation to estimate out-of-sample model performance, generating *R*^2^ and root mean squared error (RMSE). The overall approach to predictor set selection and model building is shown in Figure 1.

## Results

Of 116 participants with clinical and MRI data collected, 65 met all criteria following quality control review (including below imaging criteria) and were used for analysis. Reasons for participant exclusion included one or more of the following: insufficient available clinical data (13 participants), non-completion of DBS surgery (seven participants), insufficient electrode accuracy with respect to STN target (four participants), use of alternative electrode target (one participant), and insufficient quality of MRI data (40 participants—see “MRI methods” above). Demographics for all analyzed participants are found in Table 1. Excluded participants were clinically similar to included participants (Table S1).

Adding MRI-based predictors to pre-operative levodopa response substantially improved model performance (Table 2). Specifically, pre-operative levodopa response alone weakly predicted DBS motor response (*R*^2^ = 0.191, RMSE = 13.6). The addition of an expanded set of clinical predictors into factor selection weakly improved model fit (F = 2.13, p = 0.002) but did not improve cross-validated predictive performance (*R*^2^ = 0.150, RMSE = 14.2). The addition of MRI-based predictors also significantly improved the model *(F* = 6.56, *p* < 0.001) and improved cross-validated predictive performance *(R*^2^ = 0.313, RMSE = 12.6). FC improved cross-validated model performance slightly more than volumetrics (FC: *R*^2^ = 0.32, RMSE = 12.7; volumetric: *R*^2^ = 0.28, RMSE = 12.9). Inclusion of all potential predictors (“comprehensive model”) also improved model fit *(F* = 5.67, *p* < 0.001) and improved cross-validated predictive performance (*R*^2^ = 0.315, RMSE = 12.6). Only main effects were retained following factor selection in the comprehensive model. To avoid potential bias related to exclusion of inaccurate electrodes, analysis was repeated with inaccurate electrodes included, and findings were similar but with slightly less predictive performance for all predictor types.

**Figure 2.**
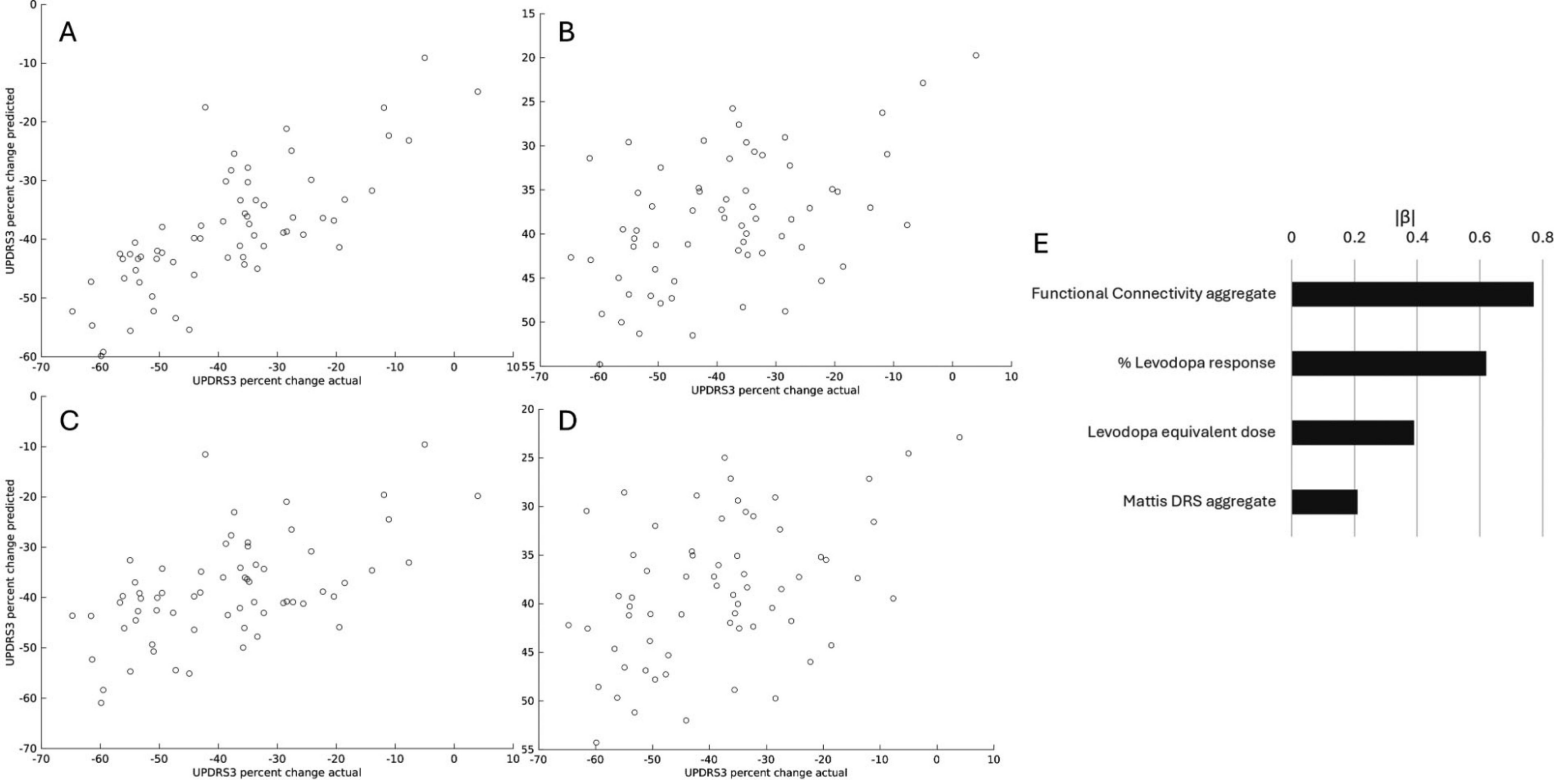
Comprehensive (optimized) model performance. Actual versus raw predicted percent motor response for (A) comprehensive model (*R*^2^ = 0.581, 95% CI 0.410–0.753) and (B) levodopa response model (*R*^2^ = 0.2335, 95% CI 0.0242–0.4428). Leave-one-out crossvalidation was then used to generate scatterplots of actual vs crossvalidated predictions for the (C) comprehensive model (*R*^2^ = 0.315) and (D) levodopa response model (*R*^2^ = 0.191). (E) Comparison of absolute value standardized regression coefficients for data types included in comprehensive model following factor selection.

Standardized regression coefficients ordered by absolute standardized coefficient (|*β*|) for the comprehensive model are presented in Table 3. Notably, pre-operative levodopa response was the single largest contributor to the comprehensive model (*β* = 0.616), followed by LEDD at time of DBS (*β* = 0.339), followed by FC principal components 7, 6, and 4 (*β* = 0.306, −0.215, −0.118, respectively). Other predictors included in the comprehensive model were Mattis DRS score (*β* = 0.118), Mattis DRS age-based percentile (*β* = −0.093), and FC principal components 2 and 1 (*β* = −0.077 and *β* = −0.057, respectively). Volumetric predictors and FC predictors other than PCA were not included in the optimized comprehensive model following automated factor selection. Relative weights of predictors in the comprehensive model aggregated by class (ie, FC) are shown in Figure 2. Aggregated FC and levodopa response were the top two predictor classes in the comprehensive model.

**Figure 3.**
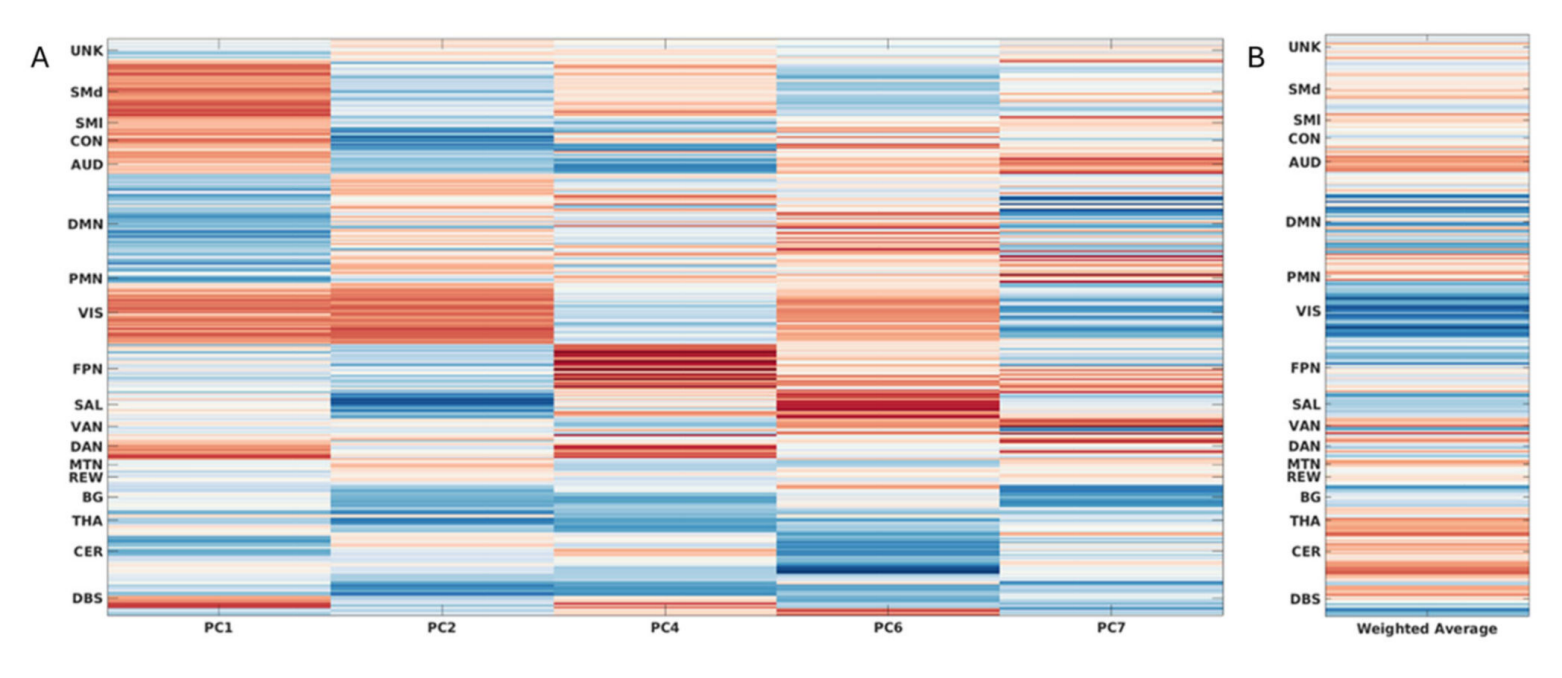
Functional connectivity profile associated with DBS response, organized by canonical resting state network by seed pair. (A) Functional connectivity loadings by principal component (PC) included in comprehensive model following factor selection. (B) Sign-adjusted weighted average (by *β*) of all included PCs in the comprehensive model, indicating overall connectivity pattern associated with DBS motor response.

Functional connectivity profiles for each principal component included in the comprehensive optimized model are presented in Figure 3, along with mean weighted FC, with *β* used as the weighting factor. In the weighted average, we observed that FC in the default mode, visual, and salience networks was associated with a greater motor response to STN DBS, while FC in the cerebellar, thalamic, and auditory networks was associated with less motor response to STN DBS.

## Discussion

We now demonstrate that inclusion of MRI-based predictors improves clinical prediction of motor response to STN DBS for PD compared with use of pre-operative levodopa motor response alone. While both FC and volumetric predictors improved predictive performance, this effect was slightly more robust for FC than for volumetric predictors, and factor selection with relaxed LASSO included only FC predictors in an optimized comprehensive model. While pre-operative levodopa response remained an important predictor in all models, its predictive performance when used alone was low in our data, congruent with other studies suggesting that this measure is insufficient to be used as a clinical predictor in isolation. The modest but significant gains in cross-validated predictive performance from adding MRI-based predictors suggest that more accurate prediction of motor benefit may be available through a multimodal approach. While the predictive power of FC for individual participants may have been limited by rs-fcMRI scan time, it is likely that greater performance for individual prediction could be obtained through future efforts at longer rs-fcMRI data collection windows, such as those obtained in precision functional mapping approaches.

Notably, all data used for prediction of post-operative outcomes in this study came from data available pre-operatively, including pre-operative clinical assessments, neuropsychological testing, and MRI data. While we excluded participants with clearly misplaced electrodes to facilitate model building, we purposefully did not include electrode location or stimulation field as a covariate due to the necessity of restricting model inputs to those available in preclinical assessments for meaningful clinical prediction. Presurgical clinical prediction likely has a theoretical upper limit in terms of the fraction of variance explained by factors which are both present and measurable prior to surgery, such as individual differences in brain structure and function, heterogeneity in PD phenotype, and factors affecting surgical risks and recovery. However, DBS outcomes are also affected by other sources of variance not amenable to pre-operative clinical prediction, such as intraoperative factors (ie, electrode placement,^50^ surgical complications^51^), and postoperative factors (i.e., programming strategy,^52^ disease progression,^53^ other medical comorbidities). The influence of these factors on motor outcomes, particularly the interaction of electrode location and stimulation field with anatomic “sweet spots” and connectivity patterns, has been described extensively in recent years.^54–56^ However, models including these factors cannot be said to be meaningfully predictive in the clinical sense. Restriction of our model to those data available pre-operatively enables its potential utility for patient selection and counseling of patients in discussions of risks and benefits of DBS for PD.

Several insights may be gained from a comparison of performance and practicality of various predictor types in routine clinical practice. First, while FC outperformed other MRI-based predictors, the absolute difference in predictive performance between FC and volumetrics was quite small. rs-fcMRI acquisition requires long scan times and intensive processing to yield usable data, while high resolution structural images suitable for volumetric analysis are generally acquired as a matter of course in presurgical planning for DBS. This may make FC impractical as a value-add for clinical prediction at many centers, while volumetric analysis would be a more implementable tool. Additionally, although LEDD and Mattis DRS were included in the optimized model, adding additional clinical predictors to levodopa response did not improve predictive power. This lack of effect of additional clinical data on model performance highlights the value of MRI predictors, and may suggest a degree of overfitting introduced by additional clinical data despite the mitigation provided by LASSO model-building. While the predictive performance of levodopa response is weak, it remains the best single clinical tool for prediction of DBS motor benefit in PD.

This study’s most notable limitation is that it is limited to data from a single center, thus potentially reducing generalizability of results. To mitigate this limitation, we conservatively estimated out of sample model performance using cross-validation. However, the single-site nature of this dataset also allowed us to perform rigorous quality control for both MRI and clinical data, with clinical raters validated against a consistent standard for the primary outcome. Although suitable datasets to test external validity of this model are not readily available, future work will include validation of this and similar models with data collected at other sites. Another limitation of this data is that since preoperative levodopa response was among the criteria used to select DBS candidates, the full variance of levodopa response may not be fully represented here, limiting study generalizability. However, patients with levodopa-resistant tremor were considered an exception to this criteria, and as a result nine included participants had levodopa responses below 30%. Furthermore, as STN DBS was the primary target used for DBS in PD at our center during the study period, participants were limited to those receiving DBS at this target. We thus did not include participants with DBS of the internal globus pallidus (GPi), and inclusion of this target will be a subject of future work.

We demonstrate that use of MRI data in prediction of DBS outcomes, particularly FC-based predictors, improves prediction of DBS outcomes over traditional use of levodopa response. Use of multimodal predictive models may improve patient selection and counseling of potential DBS candidates with PD. Continued validation, refinement, and expansion of this approach in external cohorts will be necessary prior to application in individual patients.

## Supplementary Material

supplemental table **Figure S1**. Histogram of shuffled correlation coefficients for absolute change levodopa response versus absolute DBS response following 100 Monte Carlo repetitions. Coefficient centered around *r* = 0.4 when *r* = 0 would be expected for an unbiased predictor, indicating strong bias.
**Figure S2**. Histogram of shuffled correlation coefficients for percent change levodopa response versus percent DBS response following 100 Monte Carlo repetitions. Coefficient centered around *r* = 0.05 when *r* = 0 would be expected for an unbiased predictor, indicating negligible bias.
**TABLE S1**. Comparison of clinical data between included and excluded participants. Values are reported as mean and standard deviation for continuous variables. For measures where missing data was present, n is noted. No differences were statistically significant following correction for multiple comparisons.

supplemental figures **Supplemental File 1** Spearman correlation coefficients (rho) between each predictor eligible for selection in comprehensive model.

Supporting Information

Supporting information may be found in the online version of this article.

## Figures and Tables

**TABLE 1:** Demographics and basic clinical data

Sex	41 male, 24 female
Handedness	55 right, 10 left
Age at DBS	63.5 (8.6)
Age at diagnosis	52.9 (9.4)
Years since diagnosis	10.6 (4.8)
Mattis dementia rating scale	137.8 (5.1) (*n* = 64)
Depression (z-score of GDS or BDI)	0.37 (1.0) (*n* = 61)
Mini-mental status exam	28.7 (1.6) (*n* = 64)
Levodopa daily equivalents	1726.5 (721.8)
UPDRS-III OFF-med (pre-DBS)	36.0 (8.0)
UPDRS-III ON-med (pre-DBS)	18.8 (6.4)
Levodopa response (% UPDRS-III)	−47.1 (15.1)
Bradykinesia OFF-med (pre-DBS)	17.8 (3.7)
Rigidity OFF-med (pre-DBS)	4.9 (3.3)
Tremor OFF-med (pre-DBS)	4.6 (3.8)
PIGD OFF-med (pre-DBS)	2.8 (1.6)
UPDRS-III % change (post-DBS)	−38.6 (15.2)

*Note:* Continuous variables noted as mean and standard deviation.

**TABLE 2:** Comparative model performance for each clinical and MRI-based predictor set

Predictor	Metric	
Levodopa response (% change)	*R* ^2^	0.1906
	RMSE	13.6206
Levodopa response + expanded clinical predictors	*R* ^2^	0.1490
	RMSE	14.2247
Levodopa response + volumetric predictors	*R* ^2^	0.2803
	RMSE	12.8597
Levodopa response + functional connectivity predictors	*R* ^2^	0.3066
	RMSE	12.6567
Levodopa response + all MRI-based predictors	*R* ^2^	0.3133
	RMSE	12.5553
All predictors	*R* ^2^	0.3153
	RMSE	12.5904

*Note:* All metrics were obtained via leave-one-out crossvalidation to estimate validity in out-of-sample dataset. *R*^2^ = R-squared, or fraction of data variance explained by model.

Abbreviation: RMSE, root mean square error.

**TABLE 3:** *Standardized regression coefficients* β *ordered by absolute coefficient* |β| *for comprehensive model following factor selection*

Predictor	*β*	|*β*|
Levodopa response (% change)	0.616	0.616
Levodopa equivalent daily dose	0.339	0.339
FC – principal component 7	0.306	0.306
FC – principal component 6	−0.215	0.215
FC – principal component 4	−0.118	0.118
Mattis dementia rating scale – raw score	0.118	0.118
Mattis dementia rating scale – age-adjusted percentile score	−0.093	0.093
FC – principal component 2	−0.077	0.077
FC – principal component 1	−0.057	0.057

## Data Availability

Deidentified data and code will be made available by corresponding author to qualified investigators on reasonable request.
