# Supplementary material for: Functional Connectivity and Volumetrics Improve Outcome Prediction for Deep Brain Stimulation in Parkinson’s Disease
Source: Mov Disord Clin Pract. Author manuscript; Available in PMC 2025 Nov 21. (PMC12481425; doi:10.1002/mdc3.70108)
Supplement: supplemental table — Figure S1. Histogram of shuffled correlation coefficients for absolute change levodopa response versus absolute DBS response following 100 Monte Carlo repetitions. Coefficient centered around r = 0.4 when r = 0 would be expected for an unbiased predictor, indicating strong bias. Figure S2. Histogram of shuffled correlation coefficients for percent change levodopa response versus percent DBS response following 100 Monte Carlo repetitions. Coefficient centered around r = 0.05 when r = 0 would be expected for an unbiased predictor, indicating negligible bias. TABLE S1. Comparison of clinical data between included and excluded participants. Values are reported as mean and standard deviation for continuous variables. For measures where missing data was present, n is noted. No differences were statistically significant following correction for multiple comparisons. [file NIHMS2110695-supplement-supplemental_table.docx]

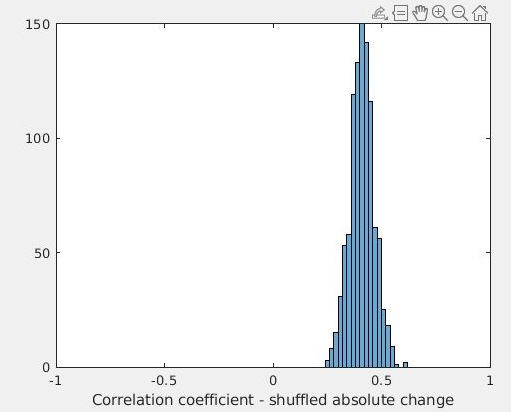


**Supplemental Figure 1: Histogram of shuffled correlation coefficients for absolute change levodopa response versus absolute DBS response following 100 Monte Carlo repetitions. Coefficient centered around r = 0.4 when r = 0 would be expected for an unbiased predictor, indicating strong bias.**


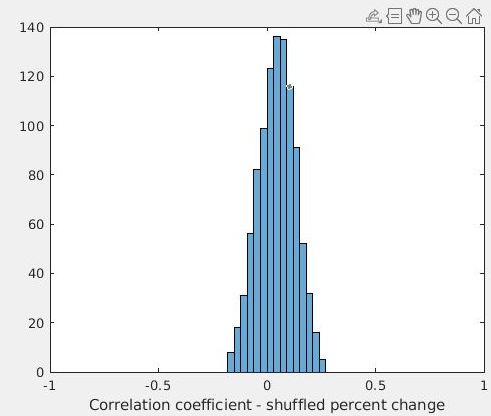


**Supplemental Figure 2: Histogram of shuffled correlation coefficients for percent change levodopa response versus percent DBS response following 100 Monte Carlo repetitions. Coefficient centered around r = 0.05 when r = 0 would be expected for an unbiased predictor, indicating negligible bias.**

|  | **Included cases (n = 65)** | **Excluded cases (n = 51)** | **p** |
| --- | --- | --- | --- |
| **Sex** | **41 male, 24 female** | **33 male, 18 female** | **0.86** |
| **Handedness** | **55 right, 10 left** | **44 right, 7 left** | **0.80** |
| **Age at DBS** | **63.5 (8.6)** | **63.3 (9.3) (n = 44)** | **0.94** |
| **Age at diagnosis** | **52.9 (9.4)** | **53.8 (9.0)** | **0.58** |
| **Years since diagnosis** | **10.6 (4.8)** | **10.8 (5.0) (n = 44)** | **0.81** |
| **Mattis Dementia Rating Scale** | **137.8 (5.1) (n = 64)** | **137.0 (5.3) (n = 49)** | **0.44** |
| **Depression (z-score of GDS or BDI)** | **0.37 (1.0) (n = 61)** | **0.54 (1.2) (n = 39)** | **0.45** |
| **Mini-Mental Status Exam** | **28.7 (1.6) (n = 64)** | **28.9 (1.3) (n = 39)** | **0.51** |
| **Levodopa daily equivalents** | **1726.5 (721.8)** | **1399.0 (623.3) (n = 44)** | **0.02** |
| **UPDRS-III OFF-med (pre-DBS)** | **36.0 (8.0)** | **38.1 (8.9) (n = 38)** | **0.22** |
| **UPDRS-III ON-med (pre-DBS)** | **18.8 (6.4)** | **19.8 (9.0) (n = 40)** | **0.55** |
| **Levodopa response (% UPDRS-III)** | **-47.1 (15.1)** | **-50.2 (18.5) (n = 38)** | **0.37** |
| **Bradykinesia OFF-med (pre-DBS)** | **17.8 (3.7)** | **18.4 (4.3) (n = 38)** | **0.43** |
| **Rigidity OFF-med (pre-DBS)** | **4.9 (3.3)** | **4.8 (4.0) (n = 38)** | **0.83** |
| **Tremor OFF-med (pre-DBS)** | **4.6 (3.8)** | **5.9 (3.7) (n = 38)** | **0.10** |
| **PIGD OFF-med (pre-DBS)** | **2.8 (1.6)** | **3.0 (1.5) (n = 38)** | **0.69** |
| **UPDRS-III % change (post-DBS)** | **-38.6 (15.2)** | **-32.8 (26.9) (n = 38)** | **0.17** |

**Supplemental Table 1: Comparison of clinical data between included and excluded participants. Values are reported as mean and standard deviation for continuous variables. For measures where missing data was present, n is noted. No differences were statistically significant following correction for multiple comparisons.**
